# Marginal and internal fit of 3D printed resin graft substitutes mimicking alveolar ridge augmentation: An *in vitro* pilot study

**DOI:** 10.1371/journal.pone.0215092

**Published:** 2019-04-15

**Authors:** C. C. Stoop, K. Chatzivasileiou, W. E. R. Berkhout, D. Wismeijer

**Affiliations:** 1 Department of Implant Dentistry and Prosthetic Dentistry, University of Amsterdam, ACTA, Amsterdam, The Netherlands; 2 Department of Oral and Maxillofacial Radiology, University of Amsterdam, ACTA, Amsterdam, The Netherlands; Medical University of Vienna, AUSTRIA

## Abstract

Recent improvements in additive manufacturing technologies may facilitate the use of customized 3D printed grafts for horizontal and vertical augmentation of the atrophic alveolar ridge. The accurate fit of such grafts could reduce the clinical treatment time and contribute optimal bone regeneration. The aim of this *in vitro* study was to evaluate the marginal and internal fit of 3D printed resin grafts as they could be used for alveolar ridge augmentation. Alveolar ridge morphologic data were derived from the Cone Beam Computed Tomography (CBCT) scans of six patients with alveolar bone defects. These data were transferred to a segmentation program to produce virtual 3D reconstructions of the alveolar ridge models. Using a Computer Aided Design (CAD) program, the alveolar bone defects were defined and customized grafts were designed and both the defects as well as the grafts generated (CAM) as 3D projects. These projects were imported into a 3D printer and were manufactured in resin. Hereafter, the grafts were fitted to the defect sites of the corresponding models and new CBCT scans were performed. Based on these scans, measurements were made at the marginal and internal part of the fitted grafts to evaluate the marginal and internal fit, respectively. The statistical analysis revealed that the mean marginal fit was significantly better (P < 0.05) than the mean internal fit. The fit of the grafts was dependent on the shape and on the size of the grafts. Specifically, the total void surface between the fitted graft and the corresponding defect site was significantly larger in the large-defect grafts than the small-defect grafts (P < 0.05). Within the limitations of the study, it could be demonstrated that it is possible to fabricate 3D printed resin grafts with acceptable fit in customized shapes, when combining CBCT scans and computer aided design and 3D printing techniques.

## Introduction

The rehabilitation of partial and fully edentulous patients with implant supported restorations has become a common treatment modality in the past few decades, showing reliable long-term results [1]. It has been demonstrated that implant supported prostheses may have a significant impact on patient’s satisfaction and masticatory performance [2]. One of the crucial aspects in implant treatment is the availability of bone volume for implant placement and the long-term support of functioning implant restorations [3].

However, the alteration of the alveolar bone dimensions following tooth loss is inevitable. Alveolar bone defects of varying sizes may occur as a result of advanced periodontitis, jaw bone cysts, tooth extractions, and dental trauma [4]. In situations of major changes in both vertical and horizontal bone dimensions, the placement of dental implants without an augmentation procedure could be very complicated or even impossible [5].

Several classifications of alveolar ridge defects can be found in the dental literature [6–8]. Most of the proposed classifications take volumetric deficiencies of the alveolar process into consideration, regardless of their aetiology, dividing them in categories of vertical, horizontal or combined defects. The state-of-the-art classification system presented by Chiapasco and Casentini focuses on the identification of alveolar bone defects as part of the planning for prosthetically driven implant rehabilitations [8]. It classifies types of bone defects as class 1 absence of bone defect till class 4 a combined horizontal and vertical bone defect. Herewith, extensive bone augmentation is mandatory and the authors postulate that implants should be placed with a delayed approach.

Nowadays, GBR is widely used for the augmentation of atrophic alveolar ridges [9, 10]. The most predictable surgical procedure for the augmentation of the severely atrophic alveolar process is the use of autogenous bone blocks [5, 11]. The bone grafts can be used as onlays to be placed on the surface of the alveolar ridge or inlays to be placed between two areas of pedicled bone with internal cancellous bone. This depends on the anatomic characteristics of the recipient site [12].

For optimal bone regeneration, bone grafts should obtain a combination of biological and mechanical properties [13]. However, the GBR technique is time-consuming and directly related to the experience and skills of the operating surgeon. The harvesting procedure is related with an elevated risk of complications such as donor morbidity and patient discomfort. After harvesting, the blocks need to be adapted to the anatomy of the recipient site and properly immobilized to ensure the re-vascularization and integration of the bone blocks. Smoothening of the sharp edges of the graft and the primary, tension-free soft tissue closure of the covering flaps are some of the aspects of the surgical procedure that can be determinant for the successful integration of the graft [14]. Furthermore, bone grafts should provide mechanical stability [15].

Another crucial aspect is the shape of the bone graft. The ideal biomaterial should be customized to fit easily on or into the corresponding bone defect and to allow proper fixation [16]. The optimal shape of the graft may come with multiple advantages in terms of: (A) Faster surgical procedures, (B) Better healing of the grafted site, (C) Reduced risk of peri- and post-operative complications, (D) Higher success rates of the bone augmentation procedure and (E) Higher patient satisfaction [17, 18].

Current bone block augmentation procedures typically require bone grafts to be manually cut, shaped, and formed during surgery. This procedure is time-consuming and is greatly dependent on the clinicians’ skill which may lead to compromised healing of the grafted site [19]. For this reason, there is a clinical need for customized biomaterials shaped to fit the patient’s bone defects [20].

Recently CAD/CAM technologies have opened new frontiers in biomedical applications. It is now possible to analyse the bone defects in 3D and to customize grafts that fit perfectly to the recipient site [21]. Specifically, recent improvements in computer guided technologies have enabled clinicians to evaluate the dimensions of bone defects in 3D prospective prior to the surgery utilizing Cone Beam Computed Tomography (CBCT) [22]. CBCT files can be transferred to specific reconstruction software, where a 3D model of the jawbones can be obtained. Finally, a custom-made graft can be designed directly on this 3D model, using CAD software [23]. The designed graft can then be sent for manufacturing to a milling machine or a 3D printer. The produced custom-made grafts are supposed to be easily adapted to the surgical site, with high accuracy. This approach can reduce the operation time and thus the discomfort of the patient [24]. For these reasons further enhancement of biomedical 3D printing technology is expected to revolutionise the field of bone augmentation.

The aim of this pilot study is to evaluate the marginal and internal fit of 3D printed resin grafts for combined vertical and horizontal alveolar bone defects *in vitro*. This study could be a proof of concept for the future development of a digital workflow in alveolar bone augmentation procedures.

## Materials and methods

### CAD/CAM fabrication of alveolar ridge models and grafts

#### Computer-aided reconstruction of alveolar ridge models and customized grafts

Cone Beam Computed Tomography (CBCT) datasets of six patients from the clinic of Oral Implantology and Prosthetic Dentistry at the Academic Centre for Dentistry Amsterdam (ACTA) were selected for this pilot study. The study protocol was approved by the Ethics Committee of the VU University Medical Center in Amsterdam (Reg.Nr.: 2018032). The Medical Research Involving Human Subjects ACT does not apply to this study. The patients gave consent to use their data, anonymously, for scientific research. All patients were partially dentate and presented with a combined vertical and horizontal defect of the maxilla (3 patients) or the mandible (3 patients). The treatment plan of these patients included implant rehabilitation in combination with an augmentation procedure involving bone blocks. The CBCT datasets of the jawbones of the patients were used for the planning of the augmentation procedures. All CBCT scans were acquired (Accuitomo 170 3D, Morita Corp., Tokyo, Japan) and exported as Digital Imaging and Communication in Medicine (DICOM) files. The initial medical imaging parameters were described in S1 Table.

The DICOM files were processed with segmentation software (Amira, FEI Corporate, Oregon, USA) to visualize sole bone structures, by changing the threshold range subjectively. After segmentation, a complete 3D reconstruction of the jaws was done. The data were exported in a stereolithographic (STL) format and defined as the dataset of the jaw models.

**Fig 1:**
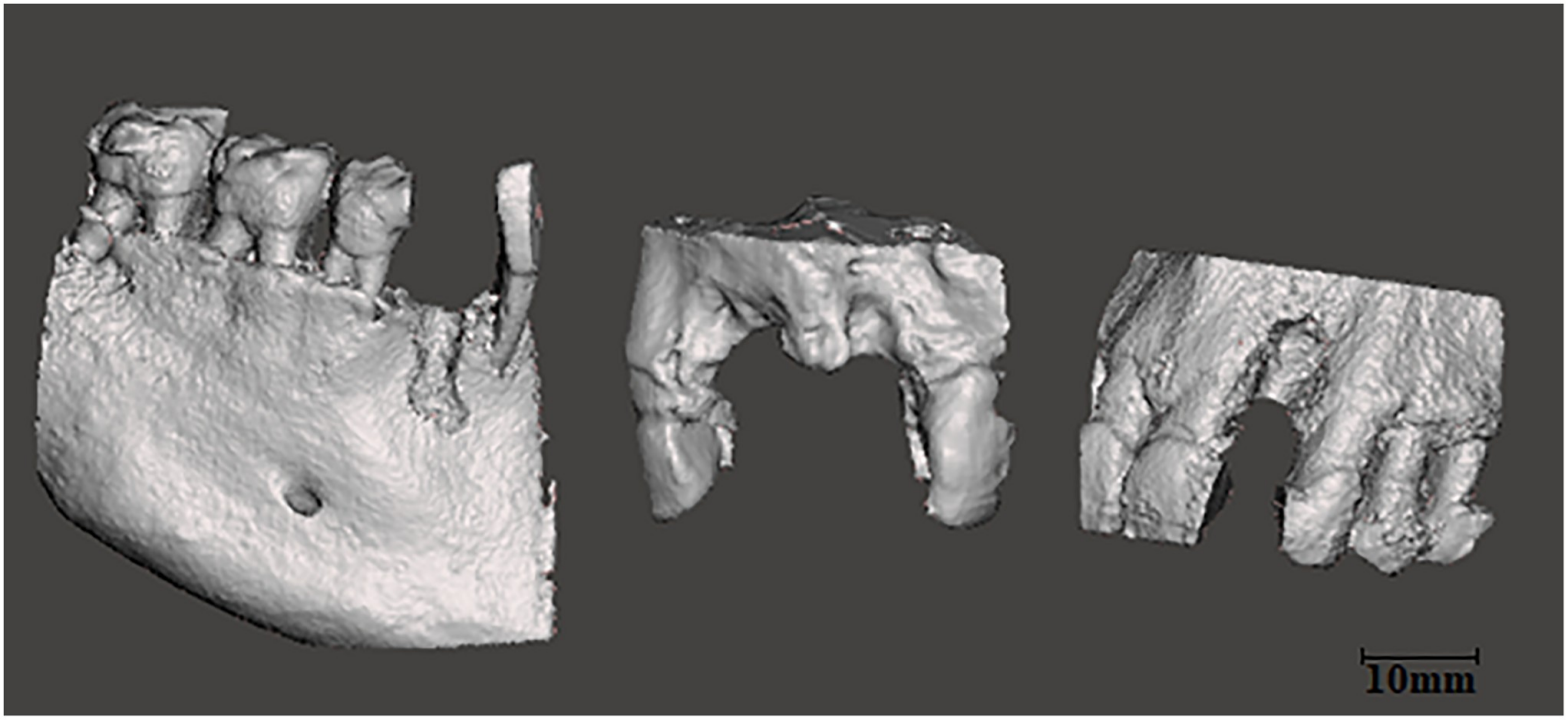
Group1: Representative lateral views of virtual 3D models with small-defects.

**Fig 2:**
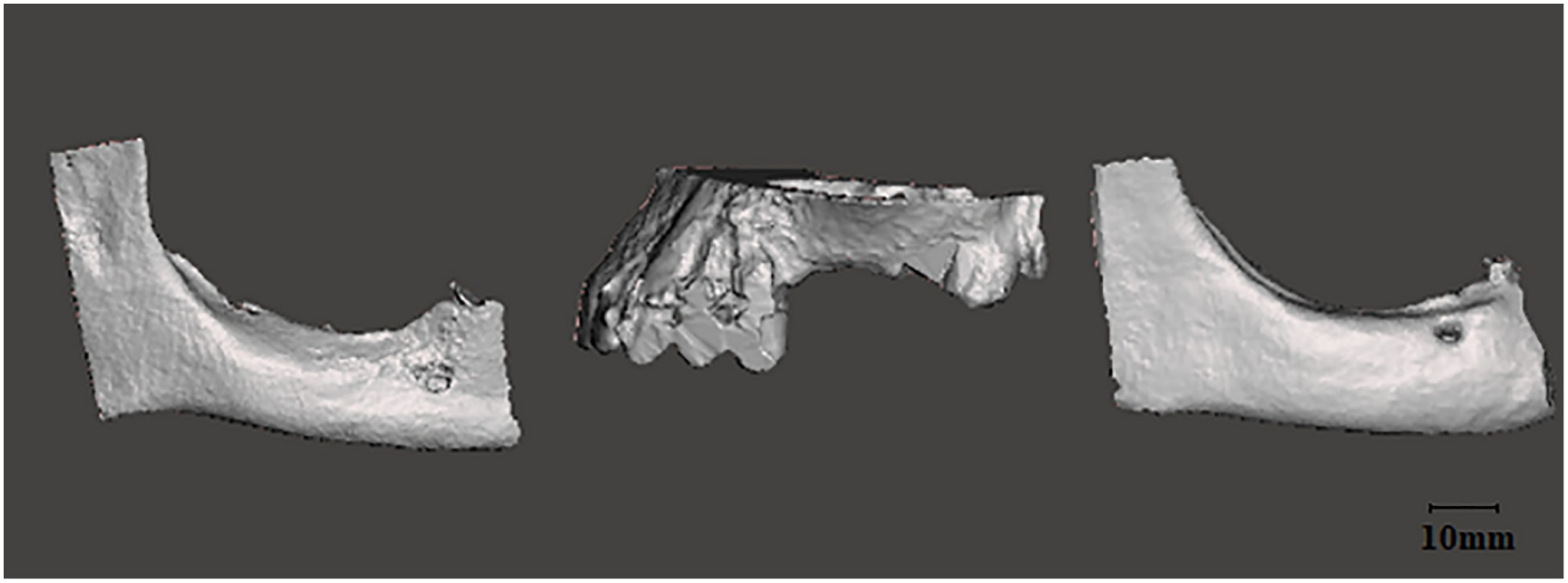
Group2: Lateral views of virtual 3D models with large-defects.

Subsequently, the models were imported into 3D designing software (Meshmixer Autodesk version 3.2, Inc., USA). The atrophic bone areas were defined separately for each jaw model in the edentulous part of the jaw. Because of the bone loss it is not possible to place an implant on this part of the jaw. The customized grafts could be manually drawn directly on the surface of the 3D projects by fabricating the original shape of the jaw. The void interface set at 0mm between model and graft. The models (n = 6) were evenly divided in two groups, according to the number of missing teeth:

1. Small-defect; defined as a defect between teeth with a maximum of two missing teeth. The grafts are L-shaped: used for the horizontal and vertical augmentation of buccal defects of the ridge (Fig 1). Corresponding grafts were shown in S1 Fig.
2. Large-defect; defined as a defect in a free ending situation with three missing teeth or more. The grafts are U-shaped: used for the horizontal and vertical augmentation of buccal and lingual/palatal defects of the ridge (Fig 2). Corresponding grafts were shown in S2 Fig.

#### 3D printing of alveolar ridge models and grafts

The three-dimensional printing of the 3D reconstructed alveolar ridge models and grafts was utilized by a desktop SLA 3D printer (Form2, Formlabs, MA, USA). The models (*n* = 6) and triplicates of the corresponding grafts (*n* = 18) were printed in high accuracy resin (Grey Resin, RS-F2-GPGR-04, Formlabs, MA, USA). Print resolution was set at 100 microns. To standardise the procedure, every graft and model was printed separately in the centre of the build platform with a 135-degree build angle. The print supports were set on the external outline to prevent them interrupting the critical internal area. After printing, the objects were washed twice in two separate 90 percent isopropyl alcohol baths (10 min each) and were postcured for 30 minutes at 45° using a 405nm light box, according to the manufacturer’s protocol.

### Cone-beam computed tomography analysis

**Fig 3:**
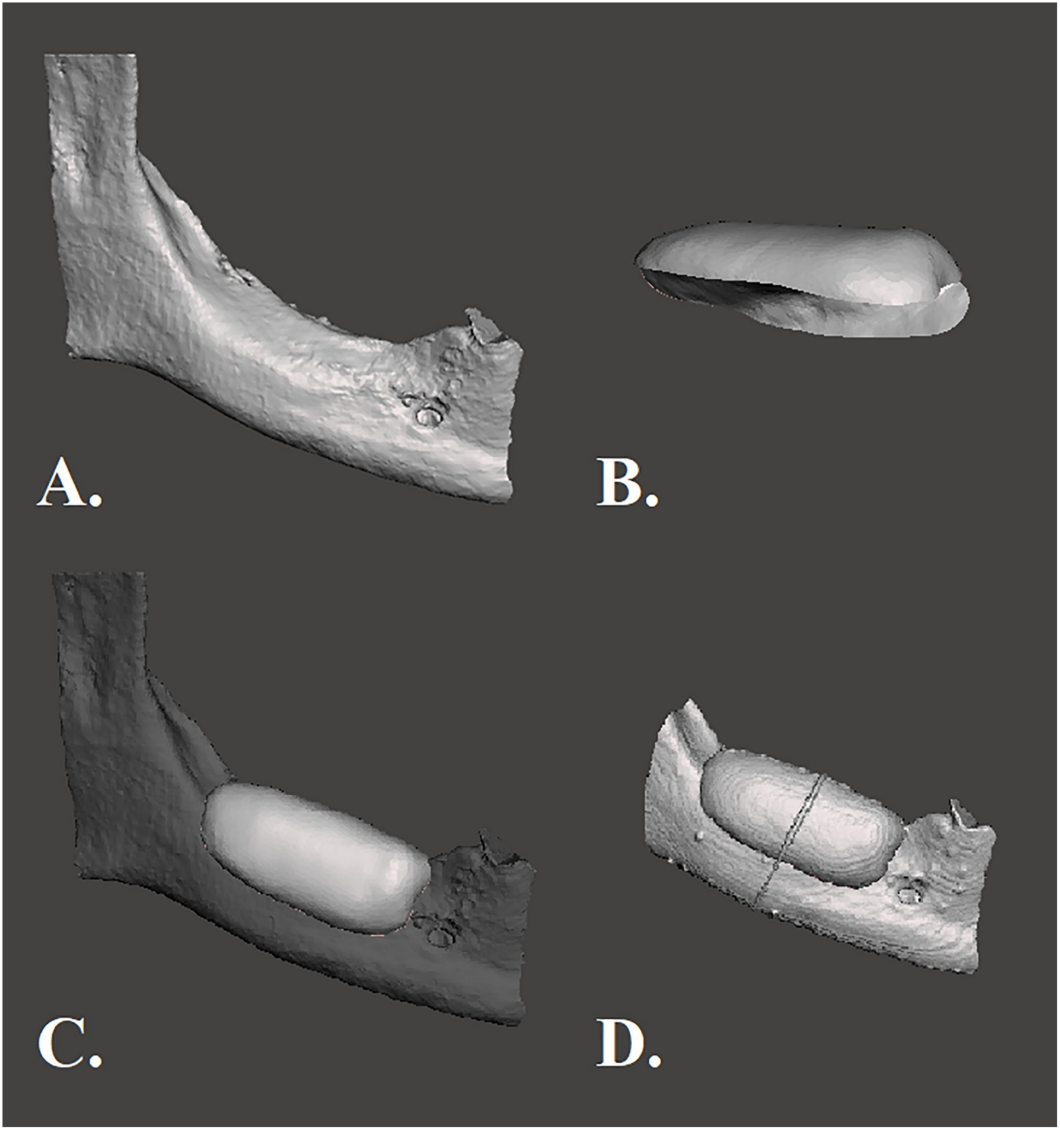
Representative 3D models of an atrophic mandible combined with one out of three large grafts. (A) Frontal view of a CAD mandibular model. (B) Lateroinferior view of a CAD large mandibular graft. (C) Frontal view of the graft fitted on the recipient site of the mandible. (D) Frontal CBCT view of the fitted graft.

The internal and external marginal fit between grafts and models were evaluated using CBCT. Three printed grafts were fitted to the corresponding defect site of their alveolar ridge model (Fig 3). The grafts were stabilized on the surface of the models with an elastic orthodontic band (Orthodontic Elastic Rubber Band, non-latex, ¼ Fox, 6.5OZ, AO, USA). The applied force was less than 0,25 N in order to allow a stable fitting of the grafts during the CBCT scanning, while avoiding, deformation or fracture of the material.

The applied forces caused by the orthodontic rubber band were measured with an orthodontic force gauge (Correx Tension Gauge, Haag-Streit A.G., Switzerland). The accuracy of the gauge was 0.05 N. Each model was scanned using CBCT (Accuitomo 170 3D, Morita Corp., Tokyo, Japan) with high voltage of 70 kV, current of 5.0 mA, voxel size of 0,125 mm, 40 x 40 mm FOV and 360° of rotation. CBCT datasets of the three small-defect models and three large-defect models, with each 3 grafts were acquired (N = 18).

The nine small grafts were visibly impeded by undercuts in the model. Therefore, the three jaw models in the small-defect group were smoothened manually with a dental mill. The undercuts were removed till the graft showed a visible fit. After this, another 9 CBCT datasets were acquired with the same procedure as the previous CBCTs. Including the second CBCTs of the small-defect group, a total of 27 CBCT datasets were acquired and used for further analysis.

#### Volume analysis

**Fig 4:**
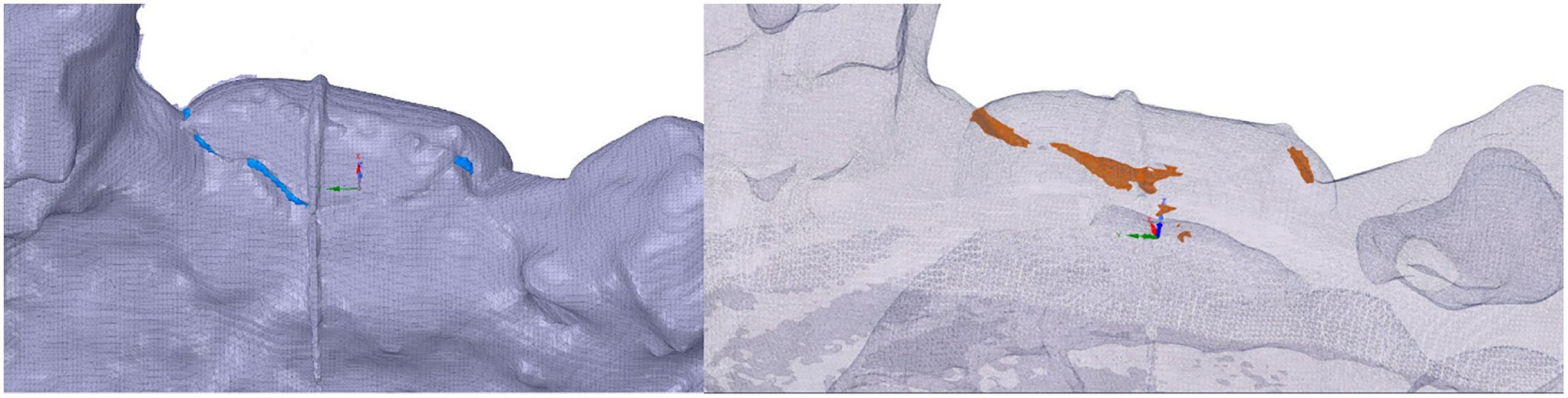
Representative virtual 3D model with graft. Blue and orange colored volume of the void between model and graft.

The acquired CBCT images were transferred to visualization software (Amira, FEI Corporate, USA) for the morphometric analysis. The data was exported in STL format and were imported in the 3D CAD design package SpaceClaim (ANSYS SpaceClaim 2017, version 18.0) for total volume measurements. A selection brush was used to disconnect the void from the outer contour of the graft. In this way only void volumes between graft and model are included. (Fig 4). The standard volume measurement tool of SpaceClaim was used to obtain the volume of the cavities in mm^3^.

#### Morphometric analysis

The CBCT images were displayed in the Amira software. The frame of each CBCT was adjusted in an axial view and centre aligned over the area of the graft. CBCT slices visualising the mesial and distal margin of each graft were defined. Based on these references the middle of the graft could be determined, separately for each model with each graft (N = 18). The dataset of the CBCT of the large-defect group and only the dataset of the second CBCT of the smoothened small-defect group was used for further analysis.

**Fig 5:**
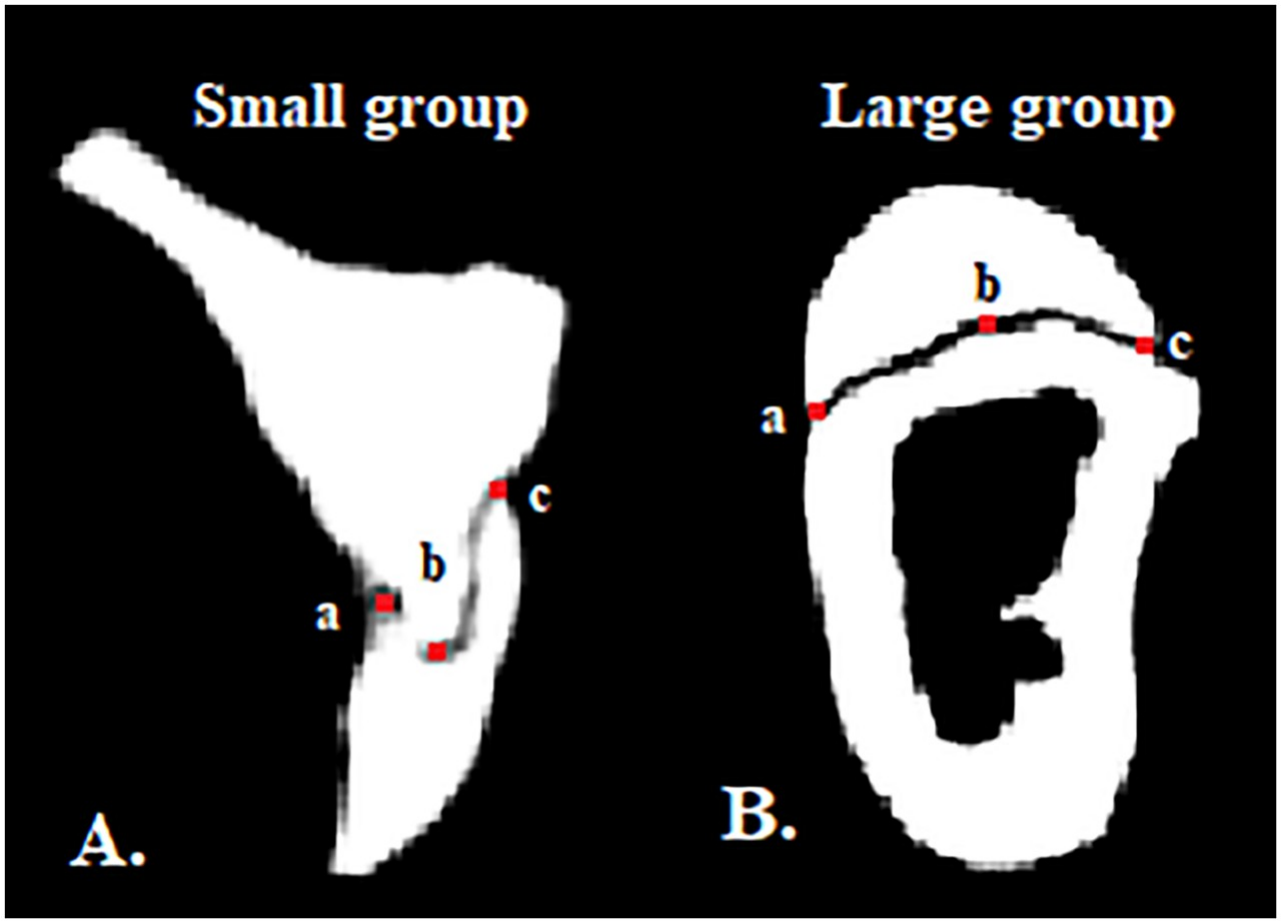
Cross-sectional slices of CBCT scans at the centre of the models with the fitted grafts. Representative slices depicting a small graft (A) and a large graft (B) fitted on the recipient defect sites of the alveolar ridge models; (a) lingual margin, (b) middle part and (c) buccal margin of the graft.

Thereafter, cross-sectional slices from the middle of each graft could be used for the analysis of the distance between graft and model, representing the fit of the graft. Consequently, zero distance or absence of void was interpreted as perfect fit between graft and model. Following landmarks were defined in each of the cross-sectional slices: (a) the lingual margin, (b) the middle part of the graft and (c) the buccal margin (Fig 5). The length of the graft surface to the model was determined as the length of the line connecting (a), (b) and (c). The internal fit was determined by the distance of the middle part of the graft to the surface of the alveolar ridge model. Accordingly, the marginal fit was determined by the distance of both the buccal and lingual marginal parts of each graft to the model. The values of the buccal and lingual marginal measurements were pooled and used for the calculation of the marginal fit of each sample. Voxel grey values from 201 and lower were included to measure the void. The voxel grey values higher than 201 were considered as voxels of the graft or model.

Furthermore, the circumference of the void area between graft and model was manually outlined and measured. To this end the STL files were loaded into another software package. All these measurements were done with linear measurement software (IC Measure, 1.3.0.605) which was calibrated to the voxel size of the CBCT.

#### Statistical analysis

Statistical analyses were carried out by independent *t*-test (IBM SPSS Statistics, Version 25.0. Armonk, NY). Pearson correlation coefficient was used to determine a correlation between void surface and graft length. Differences were considered statistically significant at *P* < 0.05.

## Results

### Volume measurements

The mean values of the void volume between graft and model of the large-defect group are shown in Table 1. The mean values of the void volume between graft and model of the small-defect group before and after smoothening the models are shown in Table 2. A significant reduction in void volume was found after smoothing the undercuts in the model (P = 0.013). Concluded, smoothing undercuts in small-defect models resulted in significant less void volume between graft and model.

**Table 1:** Void volume measurements large-defect group.

Model	1^st^ measurement CBCT void volume [mm^3^]
**4**	1,66
**5**	84,37
**6**	124,60

Mean void volume values of three grafts of each alveolar ridge model in the large-defect group.

**Table 2:** Void volume measurements small-defect group.

Model	1^st^ measurement CBCT void volume [mm^3^]	2^nd^ measurement CBCT void volume [mm^3^]
**1**	176,95	39,88
**2**	23,87	9,72
**3**	68,36	9,97

Mean void volume values of three grafts of each alveolar ridge model in the small-defect group.

### Morphometric measurements

#### Marginal and internal fit

**Fig 6:**
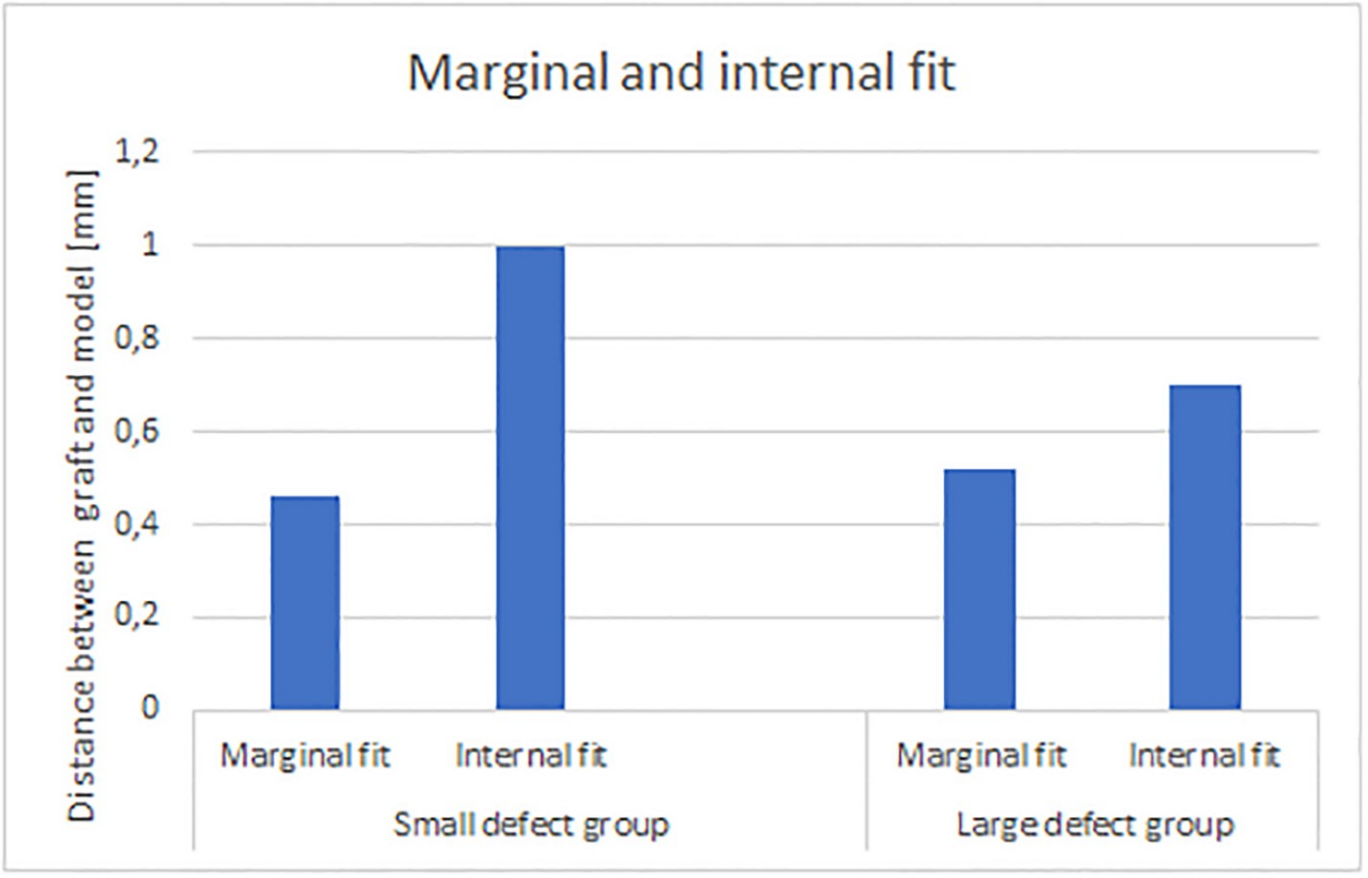
Marginal and internal fit. Bar chart presenting mean marginal and internal distances between grafts fitted on the recipient site of the alveolar ridge models. Values represent the means, n = 6.

The mean (± SD) internal fit between grafts and models was measured as 0,85 (± 0,46) mm, while the mean marginal fit showed statistically lower values as 0,49 mm (± 0,19) mm (*P* = 0.000) (Fig 6). The initial data values are shown in S2–S5 Tables.

Specifically, the mean marginal and internal fit of the grafts in the small-defect group was 0,46 ± 0,20 mm and 1,00 ± 0,60 mm, respectively. The mean marginal and internal fit in the large-defect group was 0,52 ± 0,18 mm and 0,70 ± 0,14 mm, respectively. The statistical analysis indicated no significant differences between the marginal fit in both groups, while the internal fit of the large-defect grafts was found to be significantly lower (*P* = 0.014). The mean buccal marginal fit and lingual marginal fit of the grafts in the small-defect group was 0,50 ± 0,34 mm and 0,43 ± 0,16 mm, respectively. No significant differences were found between those groups (*P* = 0.347). The mean buccal marginal fit and lingual marginal fit of the grafts in the large-defect group was 0,50 ± 0,31 mm and 0,54 ± 0,14 mm, respectively. Here, no significant differences were found between the marginal buccal and marginal lingual group (*P* = 0.566).

#### Void dimensions

The mean surface of the void was calculated as 4,23 mm^2^ ranging from 0,37 mm^2^ to 8,65 mm^2^ (Table 3). Specifically, the mean surfaces of void in the small-defect and large-defect group were 2,88 ± 2,05 mm^2^ and 5,58 ± 2,33 mm^2^, respectively. The statistical analysis revealed a significant difference between the void surfaces of the groups (*P* = 0.000). Furthermore, the circumference of the void surface of the fitted grafts was measured and was found to be significantly higher in the large-defect group (*P* = 0.000). Thereby, the void surfaces were found to have a correlation to the graft length (*P* = 0.001, Pearson correlation).

**Table 3:** Mean void-surface dimensions.

Grafts	Small-defect	Large-defect
**Marginal fit [mm]**	0,46 ± 0,20	0,52 ± 0,18
**Internal fit [mm]**	1,00 ± 0,60	0,70 ± 0,14
**Void surface [mm^2^]**	2,88 ± 2,05	5,58 ± 2,33
**Void circumference [mm]**	18,89 ± 8,79	29,11 ± 6,10
**Graft length [mm]**	10,74 ± 3,35	12,55 ± 1,45

The marginal fit in both groups was significantly lower than the internal fit (P = 0.00). Statistically significant difference was found between the lengths of the grafts in both groups (P = 0.013). For the analysis each alveolar ridge model was fitted with three grafts of same design and each graft was measured in triplicate, *n* = 18, *P* < 0.05 (independent *t*‐test).

## Discussion

In the present study, we evaluated the marginal and internal fit of customized 3D printed grafts for alveolar ridge augmentation *in vitro*. Current literature has shown only milled bone grafts and not 3D printed bone grafts, even without mentioning the values of fitting [23]. In the present study, all grafts were manufactured and analysed using a digital workflow. In contrast with other studies, the 3D printed grafts matched the defect area, large-defect grafts without the need of further manipulation and small-defect grafts after manually smoothing the undercuts located on the model. Most studies based on milled grafts showed that bone grafts or the alveolar ridge need to be prepared prior to placing the graft [25, 26].

The 3D printed grafts were divided regarding to the related bone defect into two groups: small-defect and large-defect grafts. These two types of bone grafts correspond to the classification system described by Chiapasco and Casentini as class 3 and class 4 alveolar bone defects [8]. In these kinds of severely atrophic sites, a two-staged approach is mandatory for implant rehabilitation [27]. One crucial parameter for the success of bone augmentation procedures is sufficient vascularization of the bone grafts. Vascular invasion and angiogenesis are prerequisites for new bone formation as they allow the migration of osteogenic cells, nutrients, and growth factors [28]. Insufficient vascularization could jeopardize the healing process, the integration and viability of the graft [29]. A key factor influencing proper vascular support is the fit of the graft to the bony surface, since it influences its mechanical stability and the presence of a valid interface between graft and recipient site [20].

According to the results of the present study, the mean marginal fit of the grafts was found to be better than the mean internal fit among all samples (P = 0,00). Specifically, the mean marginal fit between graft and recipient defect site ranged from 0,00 mm to 1,28 mm, while the internal fit ranged from 0,20 mm to 2,15 mm. These results support the presumption that it is possible to fabricate customized CAD/CAM grafts. Recent clinical studies, presenting the use of CAD/CAM manufactured grafts for alveolar bone augmentation, show promising results regarding the integration of the grafts and the bone gain in vertical and horizontal dimension [16, 23]. Nevertheless, the fitting of the grafts to the recipient defect sites was often just commented as perfect or closely matching [16, 23, 29]. Recently, Tarsitano et al. presented an accuracy error range of 0,4–2,46 mm after CAD/CAM mandibular reconstruction [30]. Here, the presence of marginal fit was above the limit of 0.6 mm that is proposed as biologically favourable for predictable bone fill in conventional bone block grafting procedures [31]. In oral implantology, the distance between the alveolar bone and the implant surface is described as jumping gap, which refers to the ability of bone to bridge the horizontal gap and fill the void spontaneously [32]. Araujo et al. have demonstrated that a jumping gap of less than 2 mm is needed for sufficient bone fill without the use of grafting materials [33]. Bone around implants can repair a 2mm gap, this can be compared with the non-vital surface of the graft. In this study, the void between graft and model is less than 2mm and the mean marginal fit was 0,85 mm. Thus, CAD/CAM designing and three-dimensional printing of these grafts gives promising results between the ranges which are described.

Interestingly, the defect type that the grafts were printed to fit for, seemed to influence the total void surface. The group of large-defect grafts showed significantly higher void surfaces between graft and defect site. These results could be attributed to the correlation between the size of graft and total void surface. Thus, large-defect grafts showed higher length to surface ratio when compared to small-defect grafts. These results highlight the importance of the surface structure of the bone when designing customized CAD/CAM grafts. The presence of undercuts and the absence of smooth bone surfaces, especially in fresh extraction sockets, where bone remodelling is not completed may influence the fit of the designed bone grafts [16].

Furthermore, the results of the study may have been influenced by several compromising factors. Specifically, the printing process was a standardised procedure to optimize the accuracy. The same material, printer and method was used because small differences in the placement on the building platform of the printer results in small deviations between models or grafts in terms of accuracy [34]. Thereby, the material Dental SG has a flexural strength over 50MPa. So the orthodontic rubber band, to keep the graft on the model during the scanning procedure, could not influence the material with 25N [35]. Besides this, the subjectively chosen grey values 201 and lower to measure the volume, marginal and internal void are arguable. These grey values are more reliable in CBCT than Hounsfield Units [36, 37]. But small movements of the model in the CBCT resulted in small deviant grey values. However, the device and scanning settings were equal for all models, so grey values are representative for the same density [37, 38].

Even though the results of the present study are encouraging, the proposed digital workflow needs to be clinically validated. Customized grafts may allow an excellent fit of the graft, but more important is the need to ensure its proper vascularization [39]. Poor vascular penetration could lead to early or late stage graft exposure with a high risk of graft infection and sequestration [40]. Furthermore, similar to any other conventional bone augmentation techniques, the clinical application of such grafts requires a high level of surgical skill, particularly with regard to the ability to achieve proper primary tension-free closure of the flap [27].

Except for the surgical management of CAD/CAM grafts, the biological properties of the biomaterial itself are of great importance. Till now the developed 3D printable biomaterials that could be integrated in a digital workflow are still in preclinical phase [41]. Some of the characteristics of the bone substitutes that should be analysed prior to clinical use are biocompadibility, osteoconductivity, surface porosity, surface chemistry, tissue bonding, material strength and degradation rate [42].

Lastly, the direct fit of 3D printed bone substitutes on recipient defect sites remains to be analysed in clinical setups. Inherent errors of different parts of the digital workflow, could lead to dimensional discrepancies between physical models and 3D printed grafts. Such misfits could be of clinical importance and need to be further evaluated with more models [43].

## Conclusions

This study has demonstrated the possibility to fabricate customized CAD/CAM grafts in resin. The grafts were digitally designed based on CBCT scans of partially dentate patients and manufactured using 3D printing technology. When analysed with CBCT, it was shown that the CAD/CAM resin grafts could fit on the recipient sites. The marginal fit of the grafts was better than the internal fit, while the average void dimensions seemed to be correlated to the defect type of the graft. Further *in vitro* studies with 3D printable bone substitutes are needed for the validation of this digital workflow for alveolar bone augmentation.

## Supporting information

S1 Fig The internal surface of the grafts corresponding to the defects of the small-defect group. (TIF)

S2 Fig The internal surface of the grafts corresponding to the defects of the large-defect group. (TIF)

S1 Table The CBCT device settings of the initial scans of the six patients. (PDF)

S2 Table Initial values of the void-surface dimensions of the small-defect group. (PDF)

S3 Table Mean values of the void-surface dimensions of the small-defect group. (PDF)

S4 Table Initial values of the void-surface dimensions of the large-defect group. (PDF)

S5 Table Mean values of the void-surface dimensions of the large-defect group. (PDF)
