# Supplementary figures and images for: Marginal and internal fit of 3D printed resin graft substitutes mimicking alveolar ridge augmentation: An in vitro pilot study
Source: PLoS One. 2019 Apr 15;14(4):e0215092. doi: 10.1371/journal.pone.0215092 (PMC6464328; doi:10.1371/journal.pone.0215092)

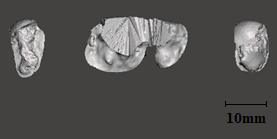

Supplement: S1 Fig — (TIF) [file pone.0215092.s001.tif]

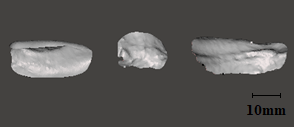

Supplement: S2 Fig — (TIF) [file pone.0215092.s002.tif]
