# Supplementary material for: Marginal and internal fit of 3D printed resin graft substitutes mimicking alveolar ridge augmentation: An in vitro pilot study
Source: PLoS One. 2019 Apr 15;14(4):e0215092. doi: 10.1371/journal.pone.0215092 (PMC6464328; doi:10.1371/journal.pone.0215092)
Supplement: S1 Table — (PDF) [file pone.0215092.s003.pdf]

Model	Voxelsize [mm]	Field of View [cm]	Highvoltage [kV]	Current [mA]	Rotation [°]
**1**	0,250	10 x 10 x 10	90	5,0	180
**2**	0,125	6 x 6 x 6	90	5,0	180
**3**	0,250	10 x 10 x 10	90	6,0	180
**4**	0,080	4 x 4 x 4	90	5,0	180
**5**	0,250	10 x 10 x 5	90	5,0	360
**6**	0,250	10 x 10 x 10	90	7,0	180
