# Supplementary material for: Marginal and internal fit of 3D printed resin graft substitutes mimicking alveolar ridge augmentation: An in vitro pilot study
Source: PLoS One. 2019 Apr 15;14(4):e0215092. doi: 10.1371/journal.pone.0215092 (PMC6464328; doi:10.1371/journal.pone.0215092)
Supplement: S2 Table — (PDF) [file pone.0215092.s004.pdf]

Small-defect grafts	Marginal fit lingual [mm]	Marginal fit buccal [mm]	Internal fit [mm]	Total surface [mm²]	Graft length [mm]	Circumference [mm]
**1**	0,388	0,388	1,863	3,99	11,25	15,45
**2**	0,225	1,275	1,488	1,11	10,46	9,95
**3**	0,225	1,088	1,875	6,32	16,53	27,96
**4**	0,550	0,338	1,763	5,14	11,57	25,20
**5**	0,475	0,200	1,975	3,36	9,37	21,45
**6**	0,388	0,375	2,150	8,65	17,19	37,01
**7**	0,488	0,500	1,388	3,51	11,01	26,17
**8**	0,375	0,538	1,663	1,93	11,95	15,47
**9**	0,538	1,163	1,788	7,05	16,06	35,61
**10**	0,638	0,263	0,713	1,64	7,33	15,40
**11**	0,525	0,700	0,925	1,62	6,18	16,26
**12**	0,400	0,413	0,675	1,02	3,77	7,44
**13**	0,375	0,750	0,763	1,36	7,17	11,71
**14**	0,413	0,525	0,650	0,89	7,70	9,23
**15**	0,500	0,363	0,700	0,65	8,14	6,67
**16**	0,550	0,850	1,075	2,58	6,98	17,96
**17**	0,813	0,700	0,725	2,56	7,35	18,07
**18**	0,700	0,538	0,900	2,00	8,74	16,35
**19**	0,338	0,000	0,550	2,54	14,18	20,02
**20**	0,225	0,488	0,638	2,80	10,46	16,09
**21**	0,513	0,463	0,563	5,18	14,21	34,26
**22**	0,425	0,438	0,500	2,41	12,50	23,41
**23**	0,488	0,738	0,513	3,20	9,47	25,86
**24**	0,500	0,338	0,550	2,99	13,04	26,16

**25**	0,088	0,038	0,200	0,37	12,94	5,72
**26**	0,188	0,000	0,175	0,69	11,25	7,95
**27**	0,300	0,000	0,350	2,14	13,22	17,19
