# Supplementary material for: Marginal and internal fit of 3D printed resin graft substitutes mimicking alveolar ridge augmentation: An in vitro pilot study
Source: PLoS One. 2019 Apr 15;14(4):e0215092. doi: 10.1371/journal.pone.0215092 (PMC6464328; doi:10.1371/journal.pone.0215092)
Supplement: S3 Table — (PDF) [file pone.0215092.s005.pdf]

	Marginal fit lingual [mm]	Marginal fit buccal [mm]	Total marginal fit [mm]	Internal fit [mm]	Total surface [mm^2]	Graft length [mm]	Circumference [mm]
**Mean values small-defect group**	0,43	0,50	0,46	1,00	2,88	10,74	18,92
