# Supplementary material for: Marginal and internal fit of 3D printed resin graft substitutes mimicking alveolar ridge augmentation: An in vitro pilot study
Source: PLoS One. 2019 Apr 15;14(4):e0215092. doi: 10.1371/journal.pone.0215092 (PMC6464328; doi:10.1371/journal.pone.0215092)
Supplement: S4 Table — (PDF) [file pone.0215092.s006.pdf]

Large-defect sampels	Marginal fit lingual [mm]	Marginal fit buccal [mm]	Internal fit [mm]	Total surface [mm²]	Graft length [mm]	Circumference [mm]
**1**	0,575	0,275	0,750	3,44	12,63	24,12
**2**	0,000	0,388	0,525	3,52	12,40	28,23
**3**	0,413	0,263	0,463	2,88	12,99	26,31
**4**	0,538	0,063	0,638	3,16	13,78	25,31
**5**	0,588	0,363	0,475	2,35	11,53	24,37
**6**	0,513	0,213	0,425	1,27	12,99	15,70
**7**	0,300	0,325	0,613	1,60	13,07	14,45
**8**	0,475	0,275	0,713	3,98	19,27	33,84
**9**	0,463	0,000	0,463	1,02	12,98	12,82
**10**	0,613	0,838	0,700	6,77	12,24	32,54
**11**	0,625	0,750	0,788	6,41	11,84	32,90
**12**	0,625	0,002	0,763	7,17	12,43	33,96
**13**	0,575	0,838	0,913	7,97	12,63	34,38
**14**	0,563	0,738	0,900	7,45	12,17	33,11
**15**	0,650	0,775	0,925	7,70	12,29	32,82
**16**	0,550	0,575	0,825	5,80	12,20	32,74
**17**	0,563	0,538	0,800	5,15	12,16	33,16
**18**	0,525	0,650	0,825	6,02	12,40	33,61
**19**	0,650	0,888	0,750	7,71	12,04	32,75
**20**	0,675	0,313	0,763	7,39	12,31	31,26
**21**	0,475	0,888	0,600	7,09	11,36	31,11
**22**	0,600	0,938	0,838	7,96	11,88	32,10
**23**	0,650	0,200	0,788	7,13	11,73	28,96
**24**	0,563	0,925	0,638	7,69	11,61	31,61

**25**	0,625	0,938	0,800	7,70	12,05	32,63
**26**	0,638	0,188	0,638	7,03	12,05	29,12
**27**	0,588	0,450	0,675	7,20	11,72	31,99
