# Supplementary material for: Marginal and internal fit of 3D printed resin graft substitutes mimicking alveolar ridge augmentation: An in vitro pilot study
Source: PLoS One. 2019 Apr 15;14(4):e0215092. doi: 10.1371/journal.pone.0215092 (PMC6464328; doi:10.1371/journal.pone.0215092)
Supplement: S5 Table — (PDF) [file pone.0215092.s007.pdf]

| | Marginal fit lingual [mm] | Marginal fit buccal [mm] | Total marginal fit [mm] | Internal fit [mm] | Total surface [$mm^2$] | Graft length [mm] | Circumference [mm] |
|---|---|---|---|---|---|---|---|
| **Mean values large-defect group** | 0,54 | 0,50 | 0,52 | 0,70 | 5,58 | 12,55 | 29,11 |
